# Quality of life in uncomplicated recurrent diverticulitis: surgical *vs*. conservative treatment

**DOI:** 10.1038/s41598-020-67094-3

**Published:** 2020-06-24

**Authors:** Viktor Justin, Selman Uranues, Hans Rabl, Abe Fingerhut

**Affiliations:** 1 Section for Surgical Research, Medical University of Graz, Graz, Austria; 2 Department of Surgery, SMZ Ost Donauspital, Vienna, Austria; 3 Department of Surgery, LKH Hochsteiermark, Leoben, Austria; 4 Department of Gastrointestinal Surgery, Ruijin Hospital, Shanghai Jiao Tong University School of Medicine, Shanghai, 20025 China

**Keywords:** Large intestine, Colon, Outcomes research

## Abstract

Elective sigmoid colectomy for recurrent uncomplicated diverticulitis remains controversial and is decided on an individual basis. Eighty patients treated conservatively (44 patients) or by elective surgery (36 patients) for recurrent uncomplicated diverticulitis were contacted and assessed for quality of life. The mean difference in quality of life scores was greater after surgery (overall + 2.14%, laparoscopic resection +4.95%, p = 0.36 and p = 0.11, respectively) as compared to conservative management. Female patients undergoing laparoscopic resection had statistically significantly higher quality of life scores than women treated conservatively (+8.98%; p = 0.049). Twenty-eight of 29 responding patients stated that they were highly satisfied and would have the operation done again. Elective sigmoidectomy is a valid treatment option for recurrent uncomplicated diverticulitis in terms of quality of life. Quality of life improved most if surgery was performed laparoscopically, especially in women.

## Introduction

Diverticular disease of the colon is a common condition in Western countries^1^. While this condition remains clinically silent in the majority of the affected population, 4% to 20% of people develop acute inflammation at least once in their lifetime^2,3^. Hospital admissions for diverticular disease, especially in patients younger than 45 years, have been increasing steadily in recent decades^1,4,5^. Historically, resection was recommended after two episodes of uncomplicated diverticulitis^6–8^, a policy based on the assumption that patients with recurrent episodes were thought to have a 30 to 60% risk of developing further recurrences, serious complications^6,9^ and reduced response to medical treatment^10^.

More recent data have challenged these findings. While recurrences after medical treatment have been described in 13.3% to 36%^11–14^ of patients, only 3% to 5% develop complicated disease as defined by Wasvary *et al*.^12,13,15^. Notably, a large population based study^11^ found a significantly higher risk for re-recurrence than that for a first recurrence (29.3% *vs*. 13.3%, p < 0.001), but without any increase in severity^16^. Consequently, prophylactic resection after two episodes to prevent complications in the immunocompetent patient is no longer advised and conservative treatment is considered to be the standard of care in uncomplicated diverticulitis^17–20^.

Up to 38% of patients report persisting abdominal complaints after conservatively treated diverticulitis^21,22^. Recurrent bouts of diverticulitis have been shown to have a negative impact on health related quality of life (HR-QOL)^23,24^. Improvements of HR-QOL and alleviation of diverticulitis-associated symptoms therefore remain among the main indications for elective interval resection^18,25^. Only one randomized study^26^ has prospectively compared HR-QOL after resection with medical treatment in recurrent diverticulitis but none, to our knowledge, has done so in uncomplicated diverticular disease.

The aim of this study was to compare HR-QOL in recurrent, uncomplicated diverticulitis after surgical *vs*. medical treatment. Secondary endpoints included complications and procedure-related patient satisfaction, as well as influence of gender on outcomes.

## Patients and Methods

Following institutional ethics committee approval (EK Medical University of Graz No. 25–290 ex 12/13), an ICD-10 (codes K57.3, K57.5, K57.9) based search was undertaken for all patients (ages 18–85 years) with at least one hospital visit (out- and/or in-patient treatment) on their records for uncomplicated diverticulitis (defined later) between 2008 and 2014 at a tertiary referral centre. To be eligible for inclusion, patients had to have had at least two or more episodes of uncomplicated diverticulitis and be aged between 18 and 85 years at study date. Patients with a history of previous colorectal surgery for indications other than diverticulitis or other previously diagnosed diseases limiting QOL (,New York Heart Association grade III-IV cardiac insufficiency, chronic obstructive pulmonary disease grade III-IV, malignancy, chronic pain syndrome and dementia) were excluded.

Diverticulitis was classified using the Hinchey classification^27^ modified by Wasvary *et al*.^15^ with corresponding computerized tomography (CT) findings as described by Kaiser *et al*.^28,29^ (Table 1).

**Table 1:** Classifications for Diverticulitis.

Modified Hinchey classification by Wasvary *et al*.^15^		CT Correlates according to Kaiser *et al*.^28^
0	Mild clinical diverticulitis	Diverticula ± colonic wall thickening
Ia	Confined pericolic inflammation or phlegmon	Colonic wall thickening with pericolic soft tissue changes
Ib	Pericolic or mesocolic abscess	Ia changes + pericolic or mesocolic abscess
II	Pelvic, distant intraabdominal, or retroperitoneal abscess	Ia changes + distant abscess (generally deep in the pelvis or interloop regions)
III	Generalized purulent peritonitis	Free gas associated with localized or generalized free fluid and possible peritoneal wall thickening
IV	Generalized faecal peritonitis	Same findings as III

Recurrent uncomplicated diverticulitis was defined as at least two bouts of diverticulitis without concomitant abscess, obstruction, fistula and/or perforation (modified Hinchey 0 and Ia^15,18,19,25,28–30^). Patients with modified Hinchey stages Ib to IV as well as those with long term complications (colonic stricture, fistula formation) were classified as having complicated disease.

Further data were obtained through a questionnaire sent by surface mail to all remaining patients. Responses were evaluated for recurrent disease and previously unknown non-inclusion factors and patients were included or excluded accordingly.

### Treatment

Initial treatment for all patients consisted in parenteral or oral antibiotics, analgesics and bowel rest. In operated individuals, elective resection of the affected colon including the rectosigmoid junction was by laparotomy or laparoscopy. The choice of surgery or continuation of conservative treatment was made by patients and attending staff on a case-by-case basis.

### Outcomes

The primary outcome was the patient-reported HR-QOL measured with the adapted Gastrointestinal Quality of Life Index (aGIQLI)^31,32^ (see supplementary information): the higher the score, the better the outcome.

Operated patients were asked to complete the Freiburger Index for Patient Satisfaction (FIPS)^33^, a questionnaire that quantifies treatment-related patient satisfaction after surgery, rates treatment-related stress, recovery, and success, and asks whether patients would have the procedure done again. Based on a 6 point scale for each item, the overall rating ranged from 1 (highest satisfaction) to 6 (lowest satisfaction). Peri-operative in-hospital complications were classified according to Clavien-Dindo^34^.

### Statistical analysis

Statistical analysis was performed with SPSS 21 (SPSS Inc., Chicago, USA). χ2 was used to compare categorical data. Student’s t test and Mann-Whitney U test were used to compare continuous variables, for normal or skewed distributions, respectively. A p < 0.05 was considered statistically significant.

Written informed consent was obtained from all participating patients. This study was conducted according to the principles of good scientific practices and the declaration of Helsinki.

## Results

**Figure 1:**
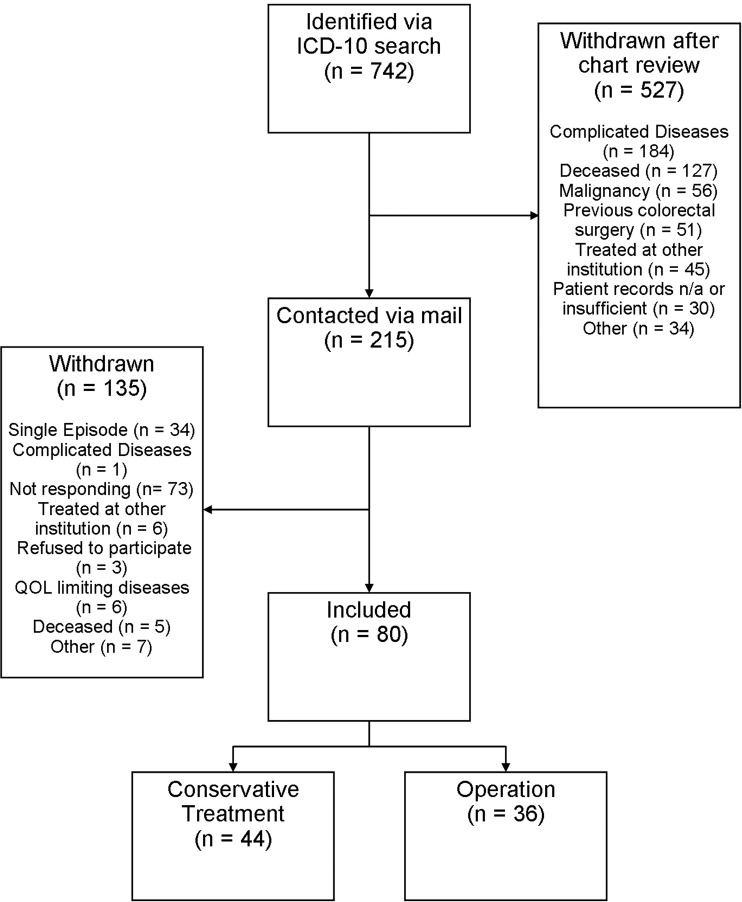
Consort flowchart n/a = not available.

Our primary search found 639 patients with at least one hospital visit (out- and/or in-patient treatment) for uncomplicated diverticulitis and an additional 103 patients operated for diverticulitis, for a total of 742 patients. After chart review, 527 patients did not meet the inclusion criteria and were excluded. The remaining 215 individuals were contacted by surface mail (Fig. 1); 126 (58.6%) responded. Patient responses were analysed again for inclusion and exclusion criteria. Ultimately 80 patients (41 men) with recurrent uncomplicated diverticulitis were included.

Patient characteristics are shown in Table 2. Forty-four (55%) patients had been treated conservatively and 36 (45%) had undergone elective surgery (24 laparoscopically). Conversion to open procedure was necessary in two patients (7.7%). There were no statistically significant differences between conservatively treated and patients undergoing resection with regard to demographics, age at first bout and number of episodes. At the time of the survey the average time since the last episode compared to the time since operation was statistically significantly longer in operated individuals (median 19 [range 1 to 55] months vs. 36 [2 to 141] months p < 0.001). None of the patients had a protective stoma at the index operation. The recurrence rate was 8.3% after surgery: two patients reported a single episode, while one reported two episodes after operation.

**Table 2:** Patient characteristics p values concern the comparison to conservative treatment ±: standard deviation BMI: Body Mass Index.

	Conservative Treatment n = 44	Surgery	
		Overall n = 36	Laparoscopy n = 24
Male	22 (50%)	19 p = 0.81	12 p = 0.60
Female	22 (50%)	17 p = 0.81	12 p = 0.58
BMI	26.1 ± 3.4	26.5 ± 3.6 p = 0.71	26.4 ± 3.3 p = 0.75
Smoking	7/30 (23.3%)	2/32 (6.3%) p = 0.06	2/22 (9.1%) p = 0.16
Fluid intake (litres/day)	1.9 ± 0.6	1.9 ± 0.6 p = 0.61	1.8 ± 0.6 p = 0.50
Age at first episode	54.3 ±10.9	58.1 ±10.4 p = 0.10	55.8 ± 11.6 p = 0.58
Months since last episode/resection (range)	19 (1–55)	36 (2–141) p < 0.001	40.5 (3–141) p < 0.001
Number of Episodes (range)	3.4 ± 1.7	3.6 ± 2.1 p = 0.86	3.7 ± 2.3 p = 0.84
Protective stoma	—	0	0

Mean aGIQLI Scores were higher in operated individuals (+2.14%; 91.70 ± 14.22 *vs*. 94.19 ± 15.33 p = 0.36; Table 3). This difference was more pronounced in individuals undergoing laparoscopic resection (+4.85%; 97.33 ± 13.28 p = 0.11). Notably, patients undergoing laparoscopic resection reported statistically significantly higher results (+10.55%; 15.02 ± 3.86 *vs*. 17.13 ± 2.35 p = 0.038) in the emotional dimension of the aGIQLI.

**Table 3:** Quality of Life p values concern the comparison to conservative treatment ±: Standard deviation. aGIQLI: adapted Gastrointestinal Quality of Life Index.

	Conservative	Surgery	
		Overall	Laparoscopy
aGIQLI total	91.7 ± 14.2	94.2 ± 15.3 p = 0.36	97.3 ± 13.3 p = 0.11
Symptoms	40.5 ± 6.1	41.0 ± 6.9 p = 0.54	42.2 ± 6.4 p = 0.27
Physical Function	18.0 ± 3.9	18.8 ± 4.6 p = 0.20	19.3 ± 4.5 p = 0.13
Emotion	15.0 ± 3.9	16.3 ± 2.9 p = 0.17	17.1 ± 2.4 p = 0.04
Social Function	14.3 ± 2.4	14.4 ± 2.5 p = 0.65	14.9 ± 1.9 p = 0.20
Therapy	3.8 ± 0.4	3.7 ± 0.8 p = 0.57	3.9 ± 0.5 p = 0.55

### Influence of gender

Apart from a statistically significantly higher BMI (25.39 ± 3.48 *vs*. 27.25 ± 3.31 p = 0.01) in males, other patient characteristics did not differ statistically significantly between the two groups (Table 4). HR-QOL scores were slightly higher in the male population.

**Table 4:** Male vs. female participants ±: Standard deviation aGIQLI: adapted Gastrointestinal Quality of Life Index BMI: Body Mass Index.

	Female N = 41	Male N = 39	p value
Total	41	39	
Surgery	19	17	
Laparoscopic	12	12	
Age at first episode (years)	56.6 ± 10.9	55.3 ± 10.8	0.61
BMI	25.4 ± 3.5	27.2 ± 3.3	0.013
Smoking (yes)	4/31 (12.9%)	5/31(16.1%)	0.72
Fluid (litre/day)	1.8 ± 0.5	2.0 ± 0.6	0.054
Number of Episodes	3.7 ± 2.1	3.3 ± 1.6	0.28
aGIQLI total	91.8 ± 14.5	93.9 ± 14.9	0.42
- Symptoms	40.6 ± 6.4	40.9 ± 6.5	0.73
- Physical Function	18.3 ± 3.9	18.4 ± 4.6	0.77
- Emotion	15.1 ± 3.7	16.1 ± 3.3	0.19
- Social Function	14.1 ± 2.5	14.6 ± 2.3	0.36
- Therapy	3.7 ± 0.6	3.8 ± 0.62	0.59

Comparing HR-QOL in conservative and laparoscopic treatment, we found a statistically significant increase (p = 0.049) after resection in women (Table 5), while men (Table 6) showed a non-significant increase (p = 0.86).

**Table 5:** Female participants p values concern the comparison to conservative treatment ±: Standard deviation aGIQLI: adapted Gastrointestinal Quality of Life Index BMI: Body Mass Index.

	Conservative N = 22	Surgery	
		Overall N = 19	Laparoscopy N = 12
Age at first episode (years)	55.6 ±11.2	57.7 ±10.7 p = 0.55	55.3 ±12.0 p = 0.94
BMI	25.9 ± 4.1	24.9 ± 2.9 p = 0.72	25.1 ± 2.4 p = 0.96
Smoking	2/14 (14.3%)	2/17 (11.8%) p = 0.84	2/12 (16.7%) p = 0.87
Fluid (litre/day)	1.8 ± 0.4	1.8 ± 0.6 p = 0.23	1.8 ± 0.7 p = 0.22
Number of episodes	3.5 ± 1.7	4.0 ± 2.5 p = 0.58	4.4 ± 3.1 p = 0.53
aGIQLI total	88.5 ± 15.6	95.7 ± 12.5 p = 0.12	98.9 ± 10.9 p = 0.049
- Symptoms	39.9 ± 6.1	41.3 ± 6.9 p = 0.52	42.6 ± 6.4 p = 0.24
- Physical Function	17.1 ± 4.3	19.8 ± 2.9 p = 0.02	20.3 ± 2.8 p = 0.014
- Emotion	14.1 ± 4.2	16.3 ± 2.5 p = 0.11	17.0 ± 2.4 p = 0.035
- Social Function	13.6 ± 2.8	14.6 ± 2.6 p = 0.24	15.1 ± 1.8 p = 0.10
- Therapy	3.8 ± 0.5	3.7 ± 0.8 p = 0.98	4.0 ± 0.0 p = 0.12

**Table 6:** Male participants p values concern the comparison to conservative treatment ±: Standard deviation aGIQLI: adapted Gastrointestinal Quality of Life Index BMI: Body Mass Index.

	Conservative N = 22	Surgery	
		Overall N = 17	Laparoscopy N = 12
Age at first episode (years)	53.0 ±10.7	58.4 ±10.3 p = 0.126	56.3 ±11.6 p = 0.43
BMI	26.2 ± 2.8	28.3 ± 3.6 p = 0.086	27.8 ± 3.6 p = 0.23
Smoking (yes)	5/16 (31.3%)	0/15 (0%)	0/11 (0%)
Fluid (litre/day)	1.8 ± 0.4	1.8 ± 0.7 p = 0.90	2.0 ± 0.6 p = 0.88
Number of Episodes	3.4 ± 1.7	3.1 ± 1.5 p = 0.696	3.0 ± 1.2 p = 0.72
aGIQLI total	94.9 ± 12.2	92.5 ± 18.3 p = 0.63	95.8 ± 15.6 p = 0.86
- Symptoms	41.1 ± 6.2	40.7 ± 7.2 p = 0.88	41.8 ± 6.6 p = 0.76
- Physical Function	19.0 ± 3.4	17.7 ± 5.9 p = 0.92	18.3 ± 5.7 p = 0.86
- Emotion	15.9 ± 3.3	16.4 ± 3.3 p = 0.71	17.3 ± 2.4 p = 0.24
- Social Function	15.0 ± 1.6	14.2 ± 2.9 p = 0.66	14.8 ± 2.1 p = 0.92
- Therapy	3.9 ± 0.3	3.7 ± 0.9 p = 0.38	3.8 ± 0.6 p = 0.48

In females the overall aGIQLI score (+8.98%; 88.50 ± 15.61 *vs*. 98.92 ± 10.93 p = 0.049), as well as the dimensions of physical function (+13.3%; 17.05 ± 4.32 *vs*. 20.25 ± 2.83 p = 0.01) and emotions (+14.55%; 14.09 ± 4.21 *vs*. 17.00 ± 2.41 p = 0.04), were statistically significantly higher in the group undergoing laparoscopic resection compared to medical treatment (Table 5).

In the male participants, the overall aGIQLI score was statistically non-significantly higher in the group undergoing laparoscopic resection (+0.7%; 94.91 ± 12.19 *vs*. 95.75 ± 15.61 p = 0.86; Table 6).

Twenty-nine patients completed the FIPS questionnaire. The FIPS showed a high level of patient satisfaction after surgery (1.79 ± 0.64; Table 7). Patients treated laparoscopically performed better than those in the open surgery group (1.67 ± 0.53 *vs*. 2.10 ± 0.82 p = 0.11). Twenty-eight of 29 patients stated that they would have the operation done again, while one patient would not.

**Table 7:** FIPS – Postoperative Patient Satisfaction p values concern the comparison to laparoscopic treatment FIPS: Freiburger Index of Patient Satisfaction ±: Standard deviation.

	Surgery overall	Laparoscopy	Open surgery
FIPS:	1.79 ± 0.64	1.66 ± 0.53	2.10 ± 0.82 p = 0.11
FIPS: *female*	1.79 ± 0.58	1.78 ± 0.43	1.80 ± 0.97 p = 0.51
FIPS: *male*	1.79 ± 0.73	1.54 ± 0.63	2.40 ± 0.63 p = 0.054

We observed an overall complication rate of 13.9% after operation (Table 8). Perioperative morbidity occurred in three patients, with one major complication (Clavien-Dindo IIIb): one patient who underwent an open sigmoidectomy after seven episodes of uncomplicated diverticulitis developed an anastomotic leak on the eighth postoperative day. A Hartmann procedure was performed, which at the time of writing had yet not been reversed. This patient also reported the lowest aGIQLI-Score (45 points) and worst FIPS (score 3.2) in the operated group.

**Table 8:** Surgical Morbidity.

	Laparoscopy	Open surgery	Total
*Short Term*			
Anastomotic Leak (Clavien-Dindo IIIb)	0	1	1
Wound Infection (Clavien-Dindo I)	1	1	2
*Long Term*			
Incisional Hernia	1	1	2
Total	2	3	5 (13.9%)

## Discussion

In this comparison of HR-QOL between patients undergoing non-operative management or elective resection for recurrent uncomplicated diverticulitis, we found that overall quality of life was better in patients after surgery, although this difference did not reach statistical significance (p = 0.36). Subgroup analysis, however, noted a statistically significantly higher HR-QOL in the aGIQLI dimension of emotional QOL in laparoscopically operated individuals. Overall, physical and emotional HR-QOL scores in female patients undergoing laparoscopic resection were statistically significantly better than in women who were treated conservatively.

To our knowledge this study is the first to directly compare quality of life after surgical and non-operative management exclusively in recurrent uncomplicated diverticulitis. Similar to the results of our study, a 2016 systematic review of patient-reported outcomes after conservative or surgical management of patients with recurrent uncomplicated diverticulitis (1858 patients in 21 studies)^35^ showed higher HR-QOL (as measured by SF-36) after laparoscopic resection (73.4; 95% confidence interval [CI], 65.7–81.1) compared to conservative treatment (58.1; 95% CI, 47.2–69.1). Additionally, they found a statistically significant reduction (9% [95% CI, 4%–14%] *vs*. 36% [95% CI, 27%–45%]) in persistent abdominal symptoms after laparoscopic resection. However, none of the studies directly compared conservative treatment to elective surgery with regard to QOL and the authors reported a substantial risk of bias (assessed with the Cochrane Collaboration’s tool for bias risk) in all studies included.

Until recently, few data have been published on HR-QOL directly comparing conservative treatment to elective resection. One study from Italy^36^ compared HR-QOL after surgical *vs*. conservative treatment in patients using SF-36 as well as using and validating the relatively new *diverticulitis quality of life*^37^ questionnaire (DV-QoL, scale 1 to 10, lowest being best QOL). Although the title of this paper indicated a population of “uncomplicated” diverticulitis, the patients in this retrospective cohort of 111 patients (97 operated, 44 managed conservatively) included those with Hinchey I and II diverticular disease. The survey was conducted via two separate telephone interviews: first, patients were asked about their pre-treatment QOL, while in the second interview their “current” (post-treatment/surgery) QOL was investigated. In terms of HR-QOL they reported superior post-treatment scores in the surgical group (mean DV-QoL 6.90 *vs*.10.61 *p* = 0.0186) as well as a statistically significant (*p* = 0.0002) improvement in pre- to post-operative QOL in surgically treated patients. However, patients in the conservatively treated group were statistically significantly older (61 ± 11 yrs. *vs*. 67 ± 14 yrs.; p = 0.006), while surgically treated patients had statistically significantly higher rates of stenosis (34% *vs*.15% p < 0.0001). Additionally, the validity of retrospective investigation of pre-treatment QOL remains debatable. 88.7% of operations were performed laparoscopically, with a conversion rate of 3.5%. This is not much different from our 67% of laparoscopically completed resections and *7*.*7*% conversions, but our sample size was small.

Van de Wall and colleagues^26^ conducted the first open labelled multicentre randomized controlled trial (DIRECT Trial) comparing conservative treatment to surgery for recurrent and ongoing complaints in left-sided diverticulitis. While this Dutch study was terminated prematurely due to increasing difficulty in patient recruitment (109 patients randomly assigned to conservative management (n = 56) or surgery (n = 53)), the authors found that the quality of life score (measured by GIQLI at 6 months) was statistically significantly higher in the surgical group (114.4 (SD 22.3) *vs*. 100.4 (SD 22.7), p < 0.0001). Similarly, VAS pain score and SF-36 (secondary endpoints) were statistically significantly better in the surgical treatment group. While overall, as in our study, patients undergoing surgery reported better quality of life scores (*vs*. conservative management), there were several differences between our study and the Dutch study. The Dutch study included both uncomplicated and complicated diverticulitis (44% of patients presented with Hinchey stages II to IV), while ours concerned only uncomplicated diverticulitis. This may explain why adverse events were observed in 34% of patients in the surgical arm of the Dutch study, while in our study, there were only 3/36. 95% of operations were performed laparoscopically with a conversion rate of 5% (*vs*. 67% and 7.7% in ours). In that study, 140 GIQLI measurements at 3 months or 6 months after randomization were available from 85 participants, whereas the expected total information from all 214 patients to be included was 428 GIQLI measurements (two completed questionnaires per patient for both time points). Therefore, the information fraction (τ) available for the interim analysis was 0.327 (140/428), which might have influenced the outcome. Lastly, their 5 year follow-up^38^ showed a 46% cross-over from initially conservatively treated patients to surgical treatment due to severe ongoing complaints.

As both the Italian^36^ and Dutch^26^ studies included patients with Hinchey stages > I, this difference makes it difficult to compare outcomes with our study.

Our results were in statistically significantly in favour of laparoscopic surgery for women. While gender related differences in treatment efficacy and HR-QOL outcome have been observed in other fields of medicine, we found only one study that addressed this topic in diverticulitis. In their study on pre- and postoperative HR-QOL after elective sigmoid resection, Pasternak *et al*.^39^ reported statistically significantly lower GIQLI scores in women than in men (110 [range 59–143, standard error of the mean (SEM) = 2.392] *vs*. 120 [range 94–139, SEM = 1.864] p < 0.001). No differences related to sex were found or analysed in the studies by Forgione^40^ or Polese^36^. We found no statistically significant difference between men and women in terms of HR-QOL. However, female patients undergoing laparoscopic resection had statistically significantly higher scores than the conservatively treated women (mean difference overall: 8.98%; physical function: +13.3%; emotions: +14.55%).

While the increase in emotional QOL might also be influenced by reduction of fear of recurrence, improvement of physical function may be attributed to the operation itself. A potential placebo effect of the surgical intervention^41^ cannot be excluded. As our study was not designed to address this question specifically, we can only hypothesize that women presenting with recurrent uncomplicated diverticulitis might particularly profit from laparoscopic resection due to higher sensitivity to the improvement of disease-related concerns and behavioural changes^36^. Another hypothesis is that women might also be more sensitive to smaller scars, but we could not verify this formally.

Twenty-eight out of 29 patients responded that they “*would have the operation done again”*. Similar satisfaction rates have been reported in studies by Pasternak *et al*. (96%)^39^ and Ambrosetti *et al*. (95%)^42^. While this question might seem trivial, we believe that it provides substantial clinical significance in favour of surgery and can serve as orientation when patients are counselled on treatment options. We nonetheless cannot rule out that the decision to operate might have influenced the perception of better QOL.

In their comparative study, Ritz *et al*.^21^ observed a 3.5% recurrence rate in surgically treated patients *vs*. 32.5% with conservative treatment (p < 0.001). A multi-centre study by Binda and colleagues^43^ similarly reported a statistically significant reduction in the risk of recurrence (5.8% *vs*. 17.2% p < 0.001) after surgery as compared to medical treatment. Other studies^39,44,45^ reported recurrence rates ranging from 6.3 to 8.7% after surgery. In the DIRECT Trial^26,38^, 13 (23%) of 56 patients in the conservative management group ultimately underwent surgery due to severe ongoing abdominal complaints during the study period, compared to 5.4***%*** of recurrence after surgery. In our study we observed postoperative episodes of diverticulitis in three patients (8.3%). We cannot, however, determine the recurrence rates in our conservatively treated patients, as there was no further follow up.

Ultimately, no matter how sound the evidence, the question asked by others^18,26^ still remains: Do the possible positive effects on quality of life outweigh the risk of surgical complications, especially anastomotic leakage and subsequent catastrophes?

Similar to others^36^, we documented one anastomotic leak, but there was no mortality. Larger studies^46,47^ have reported anastomotic leak rates of 1.4 to 1.9% and mortality rates of 0.2 to 0.76%. In the study by van de Wall *et al*.^26^, 12% of patients had anastomotic leakage, without any fatalities at 6 months follow up.

We have to acknowledge certain limitations of our study. We relied on retrospective identification of patients by chart review. While this was performed rigorously, additional information had to be obtained through a questionnaire from the individual patients. Patients might not have correctly reported all medical data, such as complication rates, thus leading to under-estimation of complications. As the original Hinchey classification is only possible after operation, and similar to one of the limitations of the Dutch randomized study^26,38^, there may have been some patients in the conservative treatment arm who might have had Hinchey II disease or higher. Moreover, the prevalence of incidental microscopic or macroscopic pericolic or colonic wall abscess found during intraoperative or histopathological analysis ranged between 12% to 25%^48,49^.

A substantial number of patients (Fig. 1) initially identified by ICD-10 search had to be excluded since after chart review, the ICD-10 codes (K57.3, K57.5 and K57.9) used for uncomplicated diverticulitis revealed “miscoding” (i.e. underestimation of disease severity *and* other gastrointestinal conditions). Retrospectively this may be attributed to changing definitions of complicated disease, the then commonly used and sometimes ambiguous Hansen-Stock classification, and/or inexact coding. The overall response rate of contacted patient was 58.8%, which lies in the described range of paper based surveys^50,51^. Additionally, our survey not only aimed at QOL but also served as a means to identify possible unknown exclusion criteria, which lead to withdrawal of more patients according to the study protocol. Comparing non-responders to responders we observed slightly younger age (60 ± 12.5 *vs*. 63 ± 11.7) and more men (62.3% *vs*. 47.8% male) in non-responders. This may be of interest as the most interesting finding of our study is the significantly higher QOL in laparoscopically operated women, of whom there are fewer among the non-responders. It should be noted that several studies^51–53^ on selection bias in questionnaire based medical studies suggest that non-responder bias due to lower response rates may not significantly impact outcomes.

While all patients received antibiotics and supportive therapy, no standard therapeutic regime was used for conservative treatment. Furthermore, sample sizes were relatively small, which may be attributed to the low number of operations performed for uncomplicated diverticulitis. HR-QOL evaluation was not obtained at a standardized point of time after the last episode/operation, but conducted at one given date, thus leading to inhomogeneity in the duration of follow up and lack of QOL baseline data. We did not specifically ask for persisting complaints or for specific causes leading to operation. With the reduction of items in the adapted GIQLI score, we might have also decreased the comparability with other published data. Lastly, the only statistically significant differences were noted in subgroup analysis.

Advances in surgical expertise have been shown to reduce conversion and complication rates in laparoscopic surgery^47^, while benefits such as faster recovery, reduced hospital stay and overall morbidity^54^ can be achieved. In agreement with the current literature, our data suggest that patients with recurrent uncomplicated diverticulitis can benefit from resection. It is not, however, our intention to challenge the predominant role of conservative treatment as such, but we would like to advocate an individualized patient centred approach, where the decision for surgery is taken after careful evaluation and information to the patient about surgical risks and benefits, in patients who are fit for surgery and in centres certified in laparoscopic colorectal surgery.

## Conclusion

Our data suggest that laparoscopic resection for recurrent uncomplicated diverticulitis is a valid option to improve quality of life with high patient satisfaction, especially in women undergoing laparoscopic resection. Due to the retrospective design and relatively small number of patients, however, these findings may be subject to potential bias, so that no definitive conclusion can be drawn.
